# Emergence and Associated Risk Factors of Vector Borne West Nile Virus Infection in Ilorin, Nigeria

**Published:** 2018-12-25

**Authors:** Olatunji Matthew Kolawole, Glory Adelaiye, Jeremiah Ikhevha Ogah

**Affiliations:** Infectious Diseases and Environmental Health Research Group, Department of Microbiology, Faculty of Life Sciences, University of Ilorin, Ilorin, Nigeria

**Keywords:** Mosquito, Vector-borne, West Nile virus, Nigeria, Prevalence

## Abstract

**Background:**

West Nile Virus (WNV) is a mosquito-borne viral pathogen that is the causative agent of West Nile fever and encephalitis. Diagnostic tools for WNV infection in Nigeria are not well established hence the current prevalence rate of WNV infection in Nigeria is unknown. We aimed to establish the serological prevalence of WNV infection in febrile patients in Ilorin, Nigeria in 2016, and to assess the risk factors associated with the acquisition of the virus.

**Methods:**

This was a cross-sectional study involving the screening of subjects presenting with febrile illnesses. While specific IgM ELISA was used to determine the seroprevalence, a closed-ended questionnaire was used to ascertain the risk factors associated with this viral infection.

**Results:**

Fifteen (7.5%) of the respondents were positive for WNV infection. Subjects living in areas in close proximity to trees and bushes (P= 0.011) and stagnant water (P= 0.001) were at a higher risk of having WNV infection. Other risk factors associated with WNV among the respondents include the none use of insecticide (P= 0.001), sitting out at night (P= 0.044), HIV positivity (P= 0.003) and having an organ transplant (P= 0.002).

**Conclusion:**

This study clearly shows a considerable WNV infection in Ilorin, with the presence of factors that can promulgate an outbreak, hence a need for further surveillance in the study area.

## Introduction

West Nile virus (WNV) is a neurotropic pathogen which belongs to the Flaviviridae family, and the causative agent of (WNV) fever and encephalitis (1, 2). The virus was first isolated in 1937 in Ugandan and named after the West Nile district (3). It was largely limited to Africa and parts of Asia until it was introduced in New York, USA in 1999 (4). Between 1999 and 2010, over 2.5 million people across the world have been infected by this virus, with about 12000 of them developing to encephalitis and 1300 deaths (5). Moreover, an outbreak of WNV infection was recorded in Greece between 2010 and 2011 which resulted in about 44 deaths.

WNV is a single strand, positive-sense RNA virus with an open reading frame of about 11kb (6). Phylogenetic analysis shows that the virus has two distinct lineages (Lineage 1 and 2) although lineages 3, 4 and 5 have also been identified. Outbreaks of WNV infection have majorly been associated with lineages 1, 2 and 5 (7, 8). While lineage 1 is transmitted both in Africa and other parts of the world, lineage 2 is restricted principal to the African continent (9). However, in 2010–2011 WNV outbreak which occurred in Greece lineage 2 was also implicated (3). WNV is a vector-borne virus which infects not just humans but birds and horses. Transmission is mainly via vectors like mosquitoes (*Culex* spp.) during the course of blood feeding (2). This is usually achieved by the vector’s injection of at least a vasodilator, platelet inhibitor and coagulation inhibitor in addition to digestive enzymes (2). Mosquito saliva in vivo plays a crucial role in the transmission of WNV (10). Apart from *Culex* spp, *Aedes* mosquitoes are also known to transmit WNV (11, 12). The human skin acts as a natural barrier to different infections, including WNV although the virus remains in the skin where inoculation occurred for 14 days after infection (13).

Majority of the infections associated with WNV in humans are asymptomatic, incubation period of the virus last amid 2 to 15 days (14, 15). Symptomatic cases of WNV fever occurs in about 20% of infected persons with flu-like symptoms emerging. Various risk factors have been associated with the development of WNV infection, they include age, hypertension, compromised immunity, organ transplantation (16–18). Factors which generally encourage contact with the vector responsible for the transmission of WNV usually increase the risk of acquisition of the infection. These factors include, proximity to bushes, presence of stagnant water, housing type and weather (19, 20).

Although very few studies have shown the prevalence of WNV in Nigeria (21), none have shown its presence in the study area. Moreover, the vectors *Culex* sp. responsible for the spread of the virus have been very common in the study area (22).

We aimed to access the prevalence of WNV infection in Nigeria and to determine factors influencing the spread of the virus and viral infection.

## Materials and Methods

### Study design/study site

This research was a hospital-based cross-sectional study of febrile malaria patients attending hospitals in Ilorin West and South of Kwara state. Patients that presented symptoms similar to malaria and typhoid fever were recruited for the study for a period of 6 months from Jan–Jun 5^th^, 2016. This study was conducted in two local government areas (Ilorin West and South) in Kwara State. Ilorin is the state capital of Kwara state in Nigeria and located on (8°30′N 5°00′E). The state is made up of about 1.5 million people and has a land mass of 32500km^2^.

### Study population/sampling technique

The study population consisted of patients that presented with symptoms of malaria and typhoid fever in Sobi specialist hospital and Civil service clinic, Ilorin, Kwara state. Febrile patients were screened and a well-structured close-ended questionnaire was administered to them after an informed consent form was dully filled and signed. Participant’s blood was taken as specimen for the necessary clinical examination.

### Inclusion criteria

- Febrile patients of all ages.
- Patients with hypertension, diabetes, chronic renal failure, and HIV.
- Patients who met the criteria and consented to partake in the study.

### Exclusion criteria

- Patients vaccinated against or recently infected with related flaviviruses.
- Patients that did not give their consent.

### Blood sample collection

Overall, 200 blood samples were collected from febrile patients who met the inclusion criteria. Five-millilitre venous blood were collected intravenously using sterile needle syringe from each study participant into a sample bottle marked with a unique number that tallied with the number on their questionnaire. Serum separation was done by centrifugation at 1600 revolutions per minute for five minutes. Serum samples were then collected and stored at −20 °C.

### Assay

The preserved sera were screened for WNV IgM antibodies using ELISA. IgM results were expressed in international unit (IU) with calibration performed against reference standards of 5.0 and 10.0IU/mL where samples with Index values ≤0.10 are negative, ≥1.0 are positive and samples that fell within the 0.11–0.99 are equivocal. IgM was performed by an indirect ELISA assay. Analysis and interpretation of results were done according to the manufacturer’s instruction (WKEA Med Supplies Corp human (WNV) IgM, China: WH-1754). The IgM used for this study has a sensitivity of 95% and specificity of 95% for West Nile virus.

### Statistical analysis

All data generated from the study was checked manually for errors in filling responses. Descriptive statistics such as mean, frequency, standard deviation, percentage, and graph were used in the discussion of the results, in order to give a lucid representation of the data analyzed. The interaction between the prevalence of WNV infection and associated risk factors were tested using χ^2^ (Chi-Square) test at 5% (P< 0.05) confidence interval. All data generated from the study was checked manually for errors in filling responses.

### Ethical approval

The approval for the study was obtained from the Ethical Review Committee of the Kwara State Ministry of Health and the informed consent was obtained from patients.

## Results

Overall, 200 febrile patients who met the inclusion criteria were enrolled. While majority of respondents (44.0%) were within the age range of 21–30yrs, female subjects accounted for a larger portion (60.0%) of the study population. Other socio-demographic characteristics of the study population are also shown in Table 1.

**Table 1.** Socio-demographic characteristics of the Study population

**Socio-demographic characteristics (n=200)**	**Study population (percentage)**
**Age**	
**11–20 yr**	25 (12.5)
**21–30 yr**	88 (44.0)
**31–40 yr**	47 (23.5)
**41–50 yr**	24 (12.0)
**61–70 yr**	8 (4.0)
**71–80 yr**	8 (4.0)
**Gender**	
**Male**	60 (30.0)
**Female**	140 (70.0)
**Tribe**	
**Yoruba**	156 (78.0)
**Hausa**	15 (7.5)
**Igbo**	22 (11.0)
**Others**	7 (3.5)
**Religion**	
**Christians**	60 (30.0)
**Muslims**	140 (70.0)
**Marital status**	
**Married**	127 (63.5)
**Single**	68 (34.0)
**Divorced**	2 (1.0)
**Widowed**	3 (1.5)
**Occupation**	
**Farmers**	7 (3.5)
**Civil servants**	53 (26.5)
**Traders**	61 (30.5)
**students**	50 (25.0)
**Others**	29 (14.5)
**Level of Education**	
**No Education**	24 (12.0)
**Primary Education**	25 (12.5)
**Secondary Education**	62 (31.0)
**Post-secondary Education**	89 (44.5)

Among the 200 respondents that participated, 15 (7.5%) were positive to (WNV) while 184 (92%) were negative with 1 (0.5%) equivocal result. A positive relationship between WNV and the socio-demographic character of the study population is shown in Table 2.

**Table 2.** Level of Education of the respondents in relation to their WNV status

**Socio-demographic characteristics**	**Positive (%)**	**Negative (%)**	**χ^2^ (P)**
**Level of Education**			29.63 (0.001) Fisher’s exact
**No Education**	6 (3.0)	18 (10.5)	
**Primary Education**	7 (3.5)	18 (10.0)	
**Secondary Education**	1 (0.5)	61 (29.0)	
**Post-secondary Education**	1 (0.5)	88 (44)	

P< 0.05 is statistically significant

Establishing the prevalence of (WNV) in relation to presence of trees and bushes around the habitat of respondents revealed of the 83 (41.5%) with trees 7 (3.5%) were WNV positive. Of 62 cases (31%) with stagnant water, 12 (6%) were WNV positive. Table 3 shows the relationship between WNV prevalence and potential risk factors associated with either the prevalence of the viral infection.

**Table 3.** Characteristics of West Nile virus Positive Individuals

**Risk factor**	**Positive (%)**
**Diabetes**	
**Yes**	0 (0)
**No**	15 (7.5)
**Hypertension**	
**Yes**	2 (1.0)
**No**	13 (6.5)
**HIV**	
**Yes**	1 (0.5)
**No**	14 (7.5)
**Organ**	
**Yes**	1 (0.5)
**No**	14 (7.5)
**Vaccination**	
**Yes**	1 (0.5)
**No**	11 (5.5)
**Not sure**	3 (1.5)
**Blood transfusion**	
**Yes**	2 (1.0)
**No**	13 (6.5)
**Surgery**	
**Yes**	2 (1.0)
**No**	13 (6.5)
**Presence of Trees and Bushes**	
**Yes**	7 (3.5)
**No**	8 (4.0)
**Presence stagnant water**	
**Yes**	12 (6.0)
**No**	3 (1.5)
**Use of Mosquito net**	
**Yes**	7 (3.5)
**No**	8 (4.0)
**Use of Insecticides**	
**Yes**	3 (1.5)
**No**	12 (6.0)
**Traveling frequently**	
**Yes**	0 (0)
**No**	15 (7.5)
**Night sit out**	
**Yes**	14 (7.0)
**No**	1 (0.5)

## Discussion

This study provides data on the serological prevalence of (WNV) among febrile subjects in Ilorin. It also describes some of the socio-demographic attributes of the participating population while establishing the presence or absence of a relationship between known risk factors of West Nile viral infection, the social demographic characteristics of the respondents and the prevalence of West Nile virus.

Young adults and middle-aged people had the highest rate in this study. This is likely due to the fact that majority of the subjects presenting with febrile illnesses at the time of sampling were within this age group. Similarly, the female respondents were more than the male ones in a ratio of 2 to 1. This was because women attending antenatal clinics of the hospitals surveyed who also had febrile illness participated in the study. This shifted the population density in favor of the female subjects. This is similar to results that determined the prevalence of WNV among febrile patients in Sudan (23).

Other demographic characteristics such as tribe and religion were largely a reflection of the study area where the study was carried out (24, 25). The most popular housing type among the respondents was single room apartments. This is a reflection of the socio-economic status of the respondents. In Nigeria, most low and medium income earners in Nigeria lived in single room apartments (26). However, there was no evidence that the housing of the subjects influenced mosquito bite or the prevalence of WNV infection. The educational level of the subjects varied extensively, while less than half of the subjects had tertiary education, 24 (12%) of them had no formal education at all.

WNV serological positivity was found among 15 respondents. This represents a 7.5% prevalence rate among the study population. This was lower than the 25% prevalence rate gotten from a similar study in Maiduguri, Nigeria (21). The difference in these prevalence rates could either be due to the difference in geographical location, prevailing climate at the time of sample collection, or differences in the type of test use. While IgM ELISA was used in this study to ascertain subjects with current or recent WNV infection, Baba et al. used plaque reduction neutralization (21). The high level of specificity of the IgM ELISA used in this study (95%), helps to eliminate largely the risk of cross-reactivity from other flaviviruses especially Dengue virus, which is common in serological test of WNV. Although, this does not entirely rule out the possibilities of cross-reactivity among other flaviviruses, which remains one of the pitfalls of using ELISA in testing for WNV.

Similar WNV prevalence rate of 9.5% and 13.2% was obtained in Kenya and Sudan (23, 27), respectively. A prevalence rate of 24% in a different part of Kenya (28) and an extremely high rate of 66% was reported (29). The differences in the results from this study and other studies could also be largely influenced by environmental conditions (30).

The distribution of WNV with respect to age among the respondents was widespread and not limited to a particular age group. Age has not been shown to be a potential risk factor in the acquisition of WNV infection. This is similar to another study (23) with a *P*-value of 0.811, where age had no role in the sero-detection of WNV. This is also in concordance with the reports in Maiduguri, Nigeria (30).

Although 11 of the 15 subjects that were positive for WNV were female, this was not statistically significant (P= 0.765) as more females participated in the study than males. In addition, gender is not a known risk factor necessary for the acquisition of WNV. This is also buttressed (27) which showed that gender did not constitute a potential risk factor for WNV acquisition among Kenyan adults. However, this is not in agreement with anotehr study (21) which noticed differences between the frequencies of positive diagnoses in males and females for WNV (P= 0.022, by Fisher’s exact test).

The educational level of the respondents showed that the prevalence of WNV reduced as their educational level increases as 13 of the 15 subjects with WNV infection had either primary education or no formal education at all. Increased level of education increases the probability of the respondents having knowledge of methods of preventing WNV infection. A statistical relationship existed between the educational level of the respondents and prevalence of WNV infection. The Centre for Disease Control (31) emphasizes the need for education (formal/informal) of the populace about WNV in order to reduce the burden of WNV Infection.

Moreover, the housing type of the respondents did not contribute statistically (P= 0.719) to the prevalence of WNV infection. Although housing type measured by roofing type could contribute to the prevalence of mosquitoes which are the vectors for WNV. The housing type did not statistically influence the prevalence of arboviruses including WNV among the respondents (27). In Chicago (32), living in older houses increased the risk of acquiring WNV infection among the studied population

Presence of trees and bushes influenced the prevalence of WNV among the respondents. Presence of trees and bushes provides habitat for mosquitoes known vectors of WNV. Environmental conditions that increase the prevalence of mosquitoes and birds increase the risk of WNV infection (33).

Similarly, stagnant water is a known factor which encourages the increase in population of mosquitoes which invariably increases the risk of WNV infection (33). WNV case-patients were significantly more likely to reside near slow-moving/stagnant water sources with heavier vegetation (20). Results from this study are similar to reports from El Paso, Texas, where WNV case-patients resided close to yards that were flooded regularly by irrigation canals (19). In Romania, the risk for WNV infection was higher among persons with mosquitoes in their homes and with flooded basements (34).

Although factors that help to reduce incidence of mosquitoes invariably helps to reduce the burden of WNV infection, the use of mosquito nets did not contribute to the prevalence of WNV among the respondents. This might be due to improper use of these mosquito nets. Various studies in Nigeria have reported various misuse of insecticide-treated nets (35).

However, the use of insecticide among the respondents played a major role in the acquisition of WNV. Preventing WNV is strongly linked to preventing mosquito bite (33) and this can be achieved through the use of insecticide. Adulticiding (36) which involves killing of the adult mosquitoes using insecticides and larviciding (36) which involves the use of insecticide has been shown to be effective in controlling the spread of mosquitoes which are the main vectors of WNV (33).

Risk for becoming infected can be influenced by time spent outdoors and decisions on whether to adopt personal precautions against mosquito bites (33), this was reflective of the results obtained in this study. In Houston, a serosurvey of homeless individuals found that time spent outdoors greatly influenced infectivity, with 12.5% of those who reported spending >12h outdoors being positive for WNV, compared to only 2% of those who reported spending ≤6h outdoors (37). In this study majority of the subjects with (WNV) infection spent considerable time outdoors.

Although diabetes and hypertension did not constitute factors influencing the acquisition of WNV in this study, factors that reduce the overall immunity of humans will help propagate the development of WNV infection. Moreover, hypertension and diabetes have been shown to be independent risk factors in the development of encephalitis from WNV infection (17). In New York City, age of ≥75 yr and diabetes mellitus were both shown to be independent risk factors for death from WNV infection (38).

Although only one of the subjects had HIV, this subject was also positive for WNV. A statistical association (P= 0.003) exists between the HIV status of the respondents and the prevalence of WNV. HIV infection is known to cause reduced immunity in individuals having the disease, thus such persons are at a higher risk of having WNV infection (39) and are regarded as a major risk factor in the development of WNV infection (9). In this study, only one of the respondents has had an organ transplant, with this same respondent being positive for WNV infection. Although a significant value was obtained from this factor, much cannot be deduced due to the very low population of subjects with organ transplant and HIV in this study. Organ transplant is a major cause of reduced immunity, which could increase the risk of developing WNV (16).

## Conclusion

The risk factors which could promulgate an outbreak of WNV infection in the study area are quite evident. The presence of WNV infection in febrile patients also show a need to establish reliable protocols for the detection of the virus in subjects to avoid wrong diagnosis, as presently, such patients with febrile illnesses are not screened for WNV. Environmental and behavioral factors which promote the spread of the virus should also be curbed.
